# Characterizing facilitators and barriers to Hypoglycemic Confidence among patients with diabetes: a qualitative descriptive study

**DOI:** 10.3389/fpsyg.2026.1772492

**Published:** 2026-06-16

**Authors:** Simin Zheng, Xuefen Lan, Qingqing Zhu, Songquan Wu, Ling Chen, Ling Zhou, Shunfei Lu, Minhua Chen, Xiaojia Zheng

**Affiliations:** 1 Department of Endocrinology, The First Affiliated Hospital of Lishui University, Lishui People’s Hospital, Lishui, China; 2 School of Medicine, Lishui University, Lishui, China; 3 Department of Nursing, The Second Affiliated Hospital of Lishui University, The Second People’s Hospital of Lishui, Lishui, China; 4 Department of Nursing, The First Affiliated Hospital of Lishui University, Lishui People’s Hospital, Lishui, China

**Keywords:** barriers, confidence, diabetes care, facilitators, hypoglycemia, qualitative study

## Abstract

**Aim:**

To explore the experiences of Hypoglycemic Confidence among Chinese adults with type 1 or 2 diabetes and identify contextual facilitators and barriers affecting its development and sustainability.

**Methods:**

A qualitative descriptive design was adopted. Semi-structured face-to-face interviews were conducted with 16 adults with type 1 or 2 diabetes from the endocrinology department of a tertiary hospital in Eastern China. Inductive content analysis of the transcribed data was guided by Bandura’s self-efficacy theory.

**Results:**

Four themes emerged: (1) direct behavioral experience; (2) vicarious observation and learning experiences; (3) verbal persuasion and external support; (4) physiological and emotional arousal.

**Conclusion:**

Healthcare professionals should pay more attention to Hypoglycemic Confidence in patients with diabetes and call for social support and safety nets, thereby facilitating psychological care, enhancing knowledge provision, and acknowledging the psychosocial comforting role of culture. These strategies may have positive implications for optimizing patient-centered diabetes care and improving the health and well-being of patients in the long term.

## 1 Introduction

Intensive glucose-lowering regimens are pivotal for mitigating long-term microvascular and macrovascular complications in diabetes mellitus (DM) (American Diabetes Association Professional Practice Committee, 2024b). However, hypoglycemia can compromise the therapeutic benefits of glycemic control (American Diabetes Association Professional Practice Committee, 2024b). Epidemiological evidence indicates that 46.5%–83.4% of patients with type 1 diabetes mellitus (T1DM) or type 2 diabetes mellitus (T2DM) experience episodes of hypoglycemia annually, including clinically silent events that prevent immediate detection (Khunti et al., 2016). Beyond increasing the risk of acute physical harm (e.g., driving accidents), hypoglycemia forms a vicious cycle consisting of psychological distress, disrupted social functioning, and diminished self-management confidence, a core determinant of sustained glycemic control (Saunders et al., 2023). Critically, the repercussions extend far beyond individual suffering. Hypoglycemia imposes substantial economic burdens on healthcare systems through emergency visits, hospitalizations, and lost productivity (Shi et al., 2021). A study on the direct medical costs of diabetic complications reported that each patient with diabetes in the United States who experienced hypoglycemia incurred an additional annual cost of $176–$16,478 (Ward et al., 2014). The International Diabetes Federation (IDF) mandates incorporating mental health as the cornerstone of diabetes care (International Diabetes Federation [IDF], 2025). This necessitates a shift from pure biomedical targets to person-centered glycemic strategies that empower patients to cope with hypoglycemia, representing a critical indicator of nursing care quality (American Diabetes Association Professional Practice Committee, 2024a). However, the prevailing interventions focus on fear of hypoglycemia (FoH) and diabetes distress (Mesa et al., 2025). This negativity bias inadvertently reinforces maladaptive behaviors, thereby compromising self-management and quality of life (Mesa et al., 2025). Crucially, illness meaning-making processes, shaped by cultural beliefs, emotional schema, and cognitive appraisals, dictate patients’ responses to hypoglycemia (Zabin et al., 2025). Nurses must master narrative competence to prioritize well-being in diabetes care. They should deeply understand patients’ experiences and deliver culturally congruent psychosocial support (International Diabetes Federation [IDF], 2025).

Hypoglycemic Confidence (HC) has recently emerged to address this gap (Lan et al., 2025b; Polonsky et al., 2017a). While validating the hypoglycemia attitudes and behaviors scale (HABS), Polonsky et al. (2015) introduced HC. Unlike FoH, which focuses on negative experiences, HC captures the positive dimension of patients’ perceived capability to prevent or manage hypoglycemic events (Polonsky, 2020). It encompasses a sense of control, safety, and comfort regarding hypoglycemia risk (Polonsky et al., 2015). Patients with higher HC levels report lower diabetes distress and anxiety. This reduction in emotional distress not only enhances their quality of life but also reduces avoidance behaviors. For instance, it lowers insulin dosage or restricts physical activity, thereby preventing metabolic instability (Polonsky et al., 2020). In summary, HC links psychological resilience, self-management efficacy, and clinical outcomes (Zabin et al., 2025). HC is strongly associated with improved self-management behaviors, better glycemic control, and enhanced psychological well-being (Lind et al., 2021; Mandrik et al., 2013). Therefore, prioritizing HC is critical to optimizing patient-centered diabetes care and enhancing the long-term health outcomes and well-being of patients with diabetes. Studies have shown that the effects of HC may be associated with socio-demographic factors, blood glucose monitoring, and psychosocial status (Demir et al., 2023; Lind et al., 2021; Menekli and Şentürk, 2022; Sönmez Sari et al., 2024). However, there are some discrepancies among previous studies. For example, Polonsky et al. (2017a) indicated that literacy and the effect of glycated hemoglobin (HbA1c) control are positively associated with HC, but Demir et al. (2023) and Alosaimi et al. (2022) did not confirm these findings. Sönmez Sari et al. (2024) conducted a mixed-methods study exploring HC levels and their influencing factors in patients with diabetes. However, the qualitative component of this study focused on exploring the experience of hypoglycemia rather than investigating the experience of HC. These reports suggest that previous findings were inconsistent. Most of these studies had a quantitative design, and their data came mainly from Western populations. Therefore, there is an urgent need for a more comprehensive and systematic exploration of the factors affecting HC to develop effective, culturally tailored intervention strategies for Chinese patients and enhance their HC.

This study is theoretically based on the self-efficacy theory (SET) of Bandura et al. (Bandura, 1977). SET posits that self-efficacy is the belief in one’s ability to execute behaviors necessary to achieve the desired outcomes. It is shaped by four primary sources of efficacy information: (1) mastery experiences (direct behavioral successes or failures); (2) vicarious experiences (observing others’ performances); (3) verbal persuasion (encouragement and feedback from others); and (4) physiological and emotional arousal (interpretation of bodily and affective states) (Bandura, 1986, 1977). These four sources interact dynamically to build, sustain, or undermine self-efficacy, determining whether individuals initiate coping behaviors, persist in the face of obstacles, and recover from setbacks (Strecher et al., 1986). Unlike models that treat confidence as a static trait, SET conceptualizes it as a dynamic, situation-specific construct that fluctuates in response to ongoing efficacy data. This perspective particularly helps understand HC because hypoglycemia management is a recurrent, self-directed behavior heavily relying on the perceived capability of patients to recognize symptoms, execute corrective actions, and navigate unpredictable episodes. By mapping patients’ experiences onto the four efficacy sources of Bandura et al., this study sought to transform descriptive themes into an explanatory framework that clarifies how and why specific experiences build or erode confidence in the management of hypoglycemia.

This study aimed to identify perceived facilitators and barriers of HC among patients with diabetes based on their views, mapping and interpreting themes via SET. Nursing professionals should integrate this theoretical perspective into the assessment and training of patients with diabetes, enabling a comprehensive understanding of modifiable influencing factors from physiological aspects to psychological aspects. This approach provides an evidence-based foundation and guides potential nurse-led intervention approaches, enhancing HC through culturally relevant entry points.

## 2 Materials and methods

### 2.1 Study design

We aimed to systematically explore the context-specific experiences of HC and identify its facilitators and barriers among Chinese adults with diabetes. A qualitative descriptive design was adopted, as this approach can particularly help capture rich, first-hand accounts when there is limited evidence (Sandelowski, 2010). Based on SET, themes were derived inductively from the data and subsequently mapped onto Bandura’s four sources of efficacy information. This dual analytical approach ensured that the results remained grounded in participant voices while achieving theoretical coherence. This study adhered to the Consolidated Criteria for Reporting Qualitative Research (COREQ) for reporting (see Appendix 1; Tong et al., 2007).

### 2.2 Study setting and recruitment

Purposive sampling was adopted to elicit and capture rich data related to the research questions. Eligible participants were recruited from the endocrinology unit of a tertiary hospital in Lishui city, Zhejiang province, China. Recruitment was conducted from September to December 2024. The inclusion criteria were as follows: (1) patients with DM who met the diagnostic criteria of the World Health Organization (WHO) in 1999 (Alberti and Zimmet, 1998); (2) age ≥ 18 years; (3) disease duration ≥ 1 year; and (4) who had experienced at least one episode of hypoglycemia in the last 6 months; and (5) was able to speak and understand Mandarin. The exclusion criteria were as follows: (1) history of psychiatric disorders; (2) poor physical condition preventing the completion of the interview; and (3) severe comorbidities or complications.

We initially aimed to recruit participants with both T1DM and T2DM to capture diverse hypoglycemia experiences. However, since T1DM accounts for only approximately 5% of diabetes cases in the Chinese adult population and T2DM accounts for the other 90%–95% (Ogle et al., 2025), only one eligible patient with T1DM was available and willing to participate during the recruitment period. Therefore, we adopted a purposive sampling strategy focused on maximizing variation in hypoglycemia experiences within the predominantly T2DM population, rather than seeking equal representation by diabetes type.

### 2.3 Data collection

Data were collected through face-to-face interviews according to the interview guide (see Table 1). Our research team developed an initial interview guide based on the study aims, which was refined during a group discussion and finalized the guide after consulting with four qualitative research experts and conducting pre-interviews with two patients. Two interviewers (Z.S. and L.X.), who were master’s and doctoral nursing students with prior experience in qualitative research, possessed in-depth knowledge of the research process and were proficient in semi-structured interviewing, conducted the interviews. Before the interview, the interviewers were introduced to the participants by a nurse (C.L.) familiar with them. They initially established a relationship of mutual trust and obtained informed consent from the participants by explaining the purpose of this study and confidentiality protocols. Ward classrooms or vacant ward were selected as interview sites to ensure a quiet and comfortable environment and to ensure the privacy of the participants. One participant withdrew for dialect-related reasons. The number of the included participants was determined based on the principle of data saturation. Data saturation is defined as a state in which new data no longer generate novel insights and merely duplicate existing information, suggesting data redundancy (Saunders et al., 2018). After interviewing 14 participants, data collection reached saturation, indicating that no new or surprising information was discovered during further data collection. Two additional participants were then interviewed to validate the identified themes. Thus, 16 participants were interviewed in total, with interview durations ranging from 20 to 55 min. The interviews were conducted in Chinese, and audios were recorded and subsequently converted verbatim into text.

**TABLE 1:** Interview guide.

Semi-structured interview guide
1. Have you ever experienced hypoglycemia? Can you tell me more about it?
2. What do you think are the reasons for your hypoglycemia episodes?
3. How did you feel after experiencing hypoglycemia? And why did you have such feelings?
4. Do you have confidence in managing your hypoglycemia effectively and preventing its recurrence? What specific measures do you take?
5. Have you received any assistance when preventing or managing hypoglycemia? Or have you encountered any difficulties?
6. What factors or forms of support could enhance your confidence in preventing or coping with hypoglycemia?

### 2.4 Data analysis

Data were transcribed verbatim in Chinese, and participants were numbered (P1–P16) to safeguard participants’ privacy. Data analysis and coding were conducted using Excel and inductive content analysis (Vears and Gillam, 2022). There are few studies on HC, and this analysis method allows for a bottom-up path to distill core categories and themes from raw interview data, thereby mitigating analytical constraints arising from the paucity of established theoretical frameworks on HC. The specific steps of the analysis were as follows:

Step 1: We reviewed all data to fully familiarize ourselves with the data content and achieve data immersion.
Step 2: We labeled statements with meaning, using indigenous concepts as much as possible to ensure cultural congruence, forming initial codes.
Step 3: For each of the identified macro-categories in the first round, the textual content was analyzed line-by-line to refine more specific fine-grained codes and form sub-categories.
Step 4: All subcategories were compared, merging similar items, adjusting discrepant items, refining or splitting ambiguous categories, and conducting a third round of coding if necessary.
Step 5: The categories and subcategories were integrated to construct a coherent thematic narrative to interpret the holistic significance of the phenomenon and reveal the connections between categories.

The first two analytical steps were conducted by Z.S. and L.X. The third and fourth steps were performed jointly by Z.S., L.X., and Z.X. All researchers contributed to the synthesis and interpretation of step 5.

We subsequently mapped inductively derived themes onto the four sources of self-efficacy information proposed by Bandura (1986). Two researchers (Z.S. and L.X.) independently classified each sub-theme by its primary efficacy source, resolving discrepancies through consensus. This deductive phase elucidated how patients’ experiences affected HC. The final themes were validated through member checking. Given that this study was disseminated in English, the use of Chinese for data analysis may raise concerns regarding methodological credibility. To mitigate this potential threat to credibility, we first transcribed the Chinese audio recordings verbatim into Chinese text, which was returned to two participants for verification and revision. They (P4 and P11) responded and confirmed that the transcribed text accurately reflected their intended meaning without any requests for changes. Therefore, no modifications were made to the transcripts based on their feedback. Subsequently, a certified professional translator (not the researchers) translated the quoted excerpts into English, followed by back-translation into Chinese by the bilingual first author (L.X.) to cross-validate against the original Chinese text and ensure semantic accuracy.

### 2.5 Rigor and reflexivity

Methodological rigor was ensured by addressing four key criteria, including credibility, dependability, confirmability, and transferability (Lincoln and Guba, 2016). Credibility was established through peer debriefing, where members of the research team reviewed and validated the identified themes. Any ambiguities or inconsistencies in the themes and sub-themes were resolved through member checking with two participants. A forward and back-translation process was implemented for bilingual translation issues to ensure data accuracy and credibility. Dependability was achieved by providing a detailed account of the research methodology, and the lead authors maintained a reflexive journal to maintain reflexivity throughout the interview and data analysis. Throughout the study, sampling decisions, revisions to the interview guide, and coding procedures were fully documented to enhance confirmability, allowing for the traceability of the analytic steps. The inclusion criteria, exclusion criteria, participant characteristics, and the data collection and analysis processes are provided in detail to support transferability, enabling readers to assess the applicability of the findings to their own contexts.

## 3 Results

### 3.1 The characteristics of participants

Sixteen participants were recruited for this study, including seven men and nine women. Their mean age was 52.00 ± 14.36 years (range: 23–71 years), and their disease duration ranged from 1 to 23 years. Fifteen patients had T2DM, and one patient had T1DM. Participants’ educational attainment ranged from elementary school to college level. Most participants (*n* = 12) were employed, while four were unemployed or retired. Most participants controlled their blood glucose through oral hypoglycemic agents and/or insulin injections, and a few controlled their blood glucose solely through lifestyle modifications. Detailed descriptive statistics for participants’ demographic and clinical characteristics are presented in Table 2.

**TABLE 2:** The characteristics of participants (*N* = 16).

Number	Gender	Age	Marital status	Educational status	Type of diabetes	Occupation	Disease duration	Diabetes management plan
P1	Female	56	Married	Primary school	T2DM	Agriculture	18	Oral medication + insulin
P2	Male	63	Married	Postgraduate	T2DM	Retired	16	Oral medication
P3	Male	70	Married	Junior high school	T2DM	Retired	7	Insulin
P4	Female	67	Married	Primary school	T2DM	Agriculture	20	Oral medication + insulin
P5	Female	65	Married	Senior high school	T2DM	Retired	2	Insulin
P6	Male	48	Married	Vocational high school	T1DM	Steeplejack	7	Insulin
P7	Male	33	Married	Senior high school	T2DM	Office worker	15	Insulin
P8	Female	43	Married	Postgraduate	T2DM	Teacher	4	Insulin
P9	Female	51	Married	Primary school	T2DM	Freelancer	1	Oral medication
P10	Male	56	Married	Junior high school	T2DM	Retired	3	Insulin
P11	Female	23	Single	Undergraduate	T2DM	Unemployed	1	Oral medication
P12	Male	51	Married	Junior high school	T2DM	Bus driver	10	Oral medication + insulin
P13	Male	71	Married	Primary school	T2DM	Agriculture	23	Insulin
P14	Female	49	Married	Senior high school	T2DM	Unemployed	1	Insulin
P15	Female	29	Single	Postgraduate	T2DM	Office worker	1	Lifestyle adjustment
P16	Female	57	Married	Primary school	T2DM	Agriculture	12	Oral medication + insulin

### 3.2 Overview of themes

Four themes were extracted from the interview data, describing participants’ confidence in coping with hypoglycemia and their lived experiences of HC. These themes were as follows: (1) direct behavioral experience; (2) vicarious observation and learning experiences; (3) verbal persuasion and external support; and (4) physiological and emotional arousal. Detailed information on the themes, sub-themes, and corresponding categories is presented in Table 3.

**TABLE 3:** Themes, sub-themes, and categories of this study.

Themes	Sub-themes	Categories
Direct behavioral experience	Positive and successful experiences	Take active measures to manage blood glucose
		Actively reflect on the causes of hypoglycemia
		Adjust strategies for raising blood glucose levels in a timely manner
		Adjust strategies for raising blood glucose levels in a timely manner
	Negative and failing experiences	Relying on family members for managing hypoglycemia
		Avoiding behavior in response to hypoglycemia
		Difficulty managing hypoglycemia at work
Vicarious observation and learning experiences	Deficiency in knowledge about hypoglycemia	Lack of recognition regarding the etiologies of hypoglycemia
		Inadequate recognition of the early symptoms of hypoglycemia
		Lack of effective first-aid skills for hypoglycemia
		Improper use of antidiabetic medications increases the risk of hypoglycemia
	The empowering role of health literacy	Summarizing practical coping skills from others’ experiences
		Taking the initiative to learn about the prevention and management of hypoglycemia
Verbal persuasion and external support	Access to professional healthcare resources	Professional guidance from medical staff
	Interpersonal support and environmental accessibility	Support and assistance from family members
		Access to resources for treating hypoglycemia
	Spiritual solace and psychological comfort	Worshiping the Buddha
		Fortune-telling
Physiological and emotional arousal	Recognition and signs of hypoglycemia-related physical symptoms	The sense of security brought by hypoglycemia awareness
		The challenges of asymptomatic hypoglycemia
	Negative emotional arousal and lack of confidence	The lack of confidence in the management of hypoglycemia
		Psychological shadow following a hypoglycemia-related adverse event
		Dilemma between intensive glycemic control and hypoglycemia risk

#### 3.2.1 Theme 1: direct behavioral experience

Direct experience is a key source of self-efficacy. In this study, participants who successfully navigated hypoglycemic episodes developed confidence by controlling their blood glucose proactively, thinking through what had caused the episode, and adjusting their strategies in a timely manner. But success was not universal. Some participants recounted unsuccessful attempts that slowly eroded their confidence. These included adopting avoidance behaviors in response to hypoglycemia, workplace situations where they could not respond in a timely manner, and situations where they relied too heavily on family members for assistance.

##### 3.2.1.1 Sub-theme 1: positive and successful experiences

Participants regarded vigilant blood glucose monitoring as an essential step for maintaining stable glucose levels. This helped them feel more confident in avoiding hypoglycemia and its complications. Those who adopted proactive strategies, including regular glucose checks, found that these practices boosted their sense of control. When their readings stayed steady, they felt reassured that they could manage any episodes of hypoglycemia in the future.

*If you know that the consequences of hypoglycemia are serious, people will take it seriously and then pay more attention to blood sugar control in general.* (P11)

*I was very confident. I would keep track of my blood sugar levels and the insulin dose I injected (get out the log sheet and show it to the researchers). My blood sugar at home usually fluctuates between 4 and 6 (mmol/L).* (P5)

Most participants were aware of the causes of their hypoglycemic episodes, including insulin dosing errors, inadequate food intake, and excessive physical activity. This understanding transformed vague fears into specific, manageable factors, fostering confidence in their ability to prevent hypoglycemia through targeted behavioral adjustments.

*Why did I have a hypoglycemic episode that time and was it hard to relieve it with glucose? I remember it was bad and it turned out to be the wrong insulin injection and that was the worst of it.* (P2)

*I was already full from lunch that day, probably because I was a little busy with work in the afternoon.* (P11)

Through repeated exposure to hypoglycemic episodes, the participants developed effective reversal strategies. These strategies ensured the initiation of prompt, appropriate, and effective responses to prevent adverse outcomes during hypoglycemic episodes.

*I used to drink a bottle of milk, after which it got better, but then it slowly stopped working. Now I think the best way is to eat chocolate and honey.* (P4)

##### 3.2.1.2 Sub-theme 2: negative and failing experiences

Some participants reported a passive response during hypoglycemic episodes because they were reluctant to the inconvenient presence of others or lacked confidence in their ability to recognize hypoglycemic symptoms.

*People around me asked if I needed help, and I turned them down. I did not want to bother anyone, so I called my son and waited for him.* (P12)

To prevent hypoglycemia, some participants adopted a precautionary approach by consuming additional food prophylactically. They self-adjusted insulin doses to maintain slightly elevated blood glucose levels, compromising optimal glycemic control for short-term safety. This avoidance-oriented mindset gradually eroded their confidence in the management of hypoglycemia.

*To prevent hypoglycemia during dialysis, I eat more porridge to keep my blood sugar high and I maintain the strength to go to dialysis.* (P4)

*My usual dose of insulin is 8 units, but I’m so afraid of hypoglycemia that I sometimes decrease it by two units and only take 6.* (P3)

Certain contextual factors can undermine the confidence of participants in the management of hypoglycemia. Some participants mentioned that when they faced periods of heavy work demands, they felt difficulties in initiating timely self-management interventions for hypoglycemia.

*I’m usually confident, but I’m not when I’m at work. Several times, I don’t know why, and I’m too busy at work to eat.* (P11)

#### 3.2.2 Theme 2: vicarious observation and learning experiences

Vicarious experience is the process through which individuals strengthen their self-efficacy by observing others successfully perform a task or by learning from indirect sources, such as education, narratives, or media. Some participants reported that knowledge gaps regarding hypoglycemia left them feeling ill-equipped to independently manage such episodes. In contrast, other participants described how they had addressed these gaps through peer learning and proactive information seeking. These efforts helped them feel more prepared and more confident in case of hypoglycemia.

##### 3.2.2.1 Sub-theme 1: deficiency in knowledge about hypoglycemia

Although adequate knowledge of hypoglycemia triggers is essential for prevention, some participants reported insufficient understanding of the underlying causes, which diminished their confidence in the management of such episodes.

*A few times, I experienced hypoglycemia in the hospital. If it happens at home, I don’t know what’s going on and what to do. I don’t know if it’s insulin injections that cause hypoglycemia.* (P8)

*I also eat a lot for lunch, so I don’t know what causes hypoglycemia.* (P11)

One participant with comorbid hypertension reported dizziness and hand tremors and could distinguish whether these symptoms originated from elevated blood pressure or hypoglycemia. In contrast, other participants could not recognize hypoglycemic episodes when experiencing symptoms such as hunger or diaphoresis, leaving them less confident to take appropriate corrective actions.

*I sometimes experience sudden sweats, and I have mild hand tremors. I don’t know if it’s because of age-related weakness or hypertension. During these episodes, I didn’t know if it was hypoglycemia, and I didn’t know if I should lower my blood pressure or take a sugar supplement for fear of making an incorrect decision.* (P13)

Although some participants recognized hypoglycemic symptoms, they adopted overcompensatory or ineffective glucose-elevating interventions. These inappropriate and potentially unsafe behaviors compromised glycemic control and eroded their confidence in the management of hypoglycemic episodes.

*I don’t know what to eat to raise my blood sugar faster when hypoglycemia occurs, or how much to eat, so I just keep eating and eat a lot every time.* (P1)

*I only drank water when I was hypoglycemic, but the nurse told me that it won’t help.* (P9)

Due to insufficient knowledge about antidiabetic medications, participants were uncertain about insulin type selection during self-administration, leading to incorrect dosing and hypoglycemia. Furthermore, some participants did not lower medication doses despite fasting or reduced food intake. These medication-related lapses directly undermined their confidence in the self-management of diabetes.

*Once, I injected the wrong insulin. I was supposed to take four shots of insulin, uh, three of the short-acting insulin and one of the long-acting insulin. I injected the short-acting instead of the long-acting at night.* (P2)

*I hadn’t eaten since I had a cold that day, but I took my drugs as usual. Then, I went hypoglycemic.* (P4)

##### 3.2.2.2 Sub-theme 2: the empowering role of health literacy

Participants who learned from experience accumulated preventive and coping skills, enhancing their confidence and proficiency in effective and efficient responses.

*After all these years, I seem to have a bit of experience with dealing with hypoglycemia.* (P4)

Health literacy, the ability to access, comprehend, and apply health information, enabled participants to achieve better glycemic control and greater confidence in addressing the challenges caused by hypoglycemia.

*I follow a diabetes specialist and check the educational content he posts every day, so I’ve read a lot and learned a lot about diabetes and hypoglycemia*. (P5)

*Now, I read other people’s experiences with hypoglycemia shared on various websites and watch science popularization content, which has taught me many practical ways to manage hypoglycemia.* (P15)

#### 3.2.3 Theme 3: verbal persuasion and external support

Verbal persuasion, whether from healthcare professionals or family members, strengthens self-efficacy by affirming an individual’s capability to manage challenging situations. Participants in this study described how clinical guidance, emotional backing from relatives, and accessible emergency resources each contributed to their confidence in handling hypoglycemic episodes. Beyond these tangible supports, some participants also drew psychological comfort from culturally embedded spiritual practices, such as Buddhist prayer or fortune-telling, which served as an additional layer of emotional reassurance.

##### 3.2.3.1 Sub-theme 1: access to professional healthcare resources

In some participants, structured training regarding hypoglycemia and clinical guidance from healthcare professionals enhanced the sense of safety and confidence in the management of hypoglycemic episodes. Some participants also reported enhanced safety when using continuous glucose monitoring (CGM), as real-time glucose readings provided a sense of control over fluctuations in blood glucose levels.

*I usually inject 5 units of insulin, but I still have hypoglycemic episodes. I can call the nurses’ station and ask them for advice. I trust them more.* (P6)

*I feel so confident with this monitoring (CGM) that I can’t live without it now!* (P2)

Some participants reported their experience of receiving traditional Chinese medicine (TCM) interventions. Various TCM interventions, such as herbal medicine and acupuncture, optimized their blood glucose management ability and reduced the frequency of hypoglycemia. In addition, they reported that TCM enhanced their confidence in the management of hypoglycemia.

*The Chinese medicine practitioner said I have a spleen deficiency. I receive fire cupping therapy every month, and my blood glucose has been better controlled during this period.* (P1)

*A friend previously recommended a Chinese medicine doctor to me. I consulted the doctor and informed him that I had frequent hypoglycemic episodes. The doctor advised me to take Chinese herbal medicine to regulate my body, and also recommended acupuncture and acupressure. Following these interventions, I experienced fewer hypoglycemic episodes.* (P15)

##### 3.2.3.2 Sub-theme 2: interpersonal support and environmental accessibility

During severe hypoglycemic episodes, participants reported increased reliance on family members for physical assistance, which enhanced their confidence in the management of hypoglycemia.

*Sometimes hypoglycemia can kill you. If hypoglycemia leads to a coma, my family can immediately notice if I faint. They know that I may be hypoglycemic, and they have experience with that.* (P4)

Emotional support and practical assistance from partners positively affected the psychological health and behavior of the participants, enhancing their self-efficacy in the management of hypoglycemia.

*He is more concerned about my blood glucose than I am. Every time I check my blood sugar, we both check it together, and he records it for me afterward. My husband is really good.* (P5)

Participants reported that having a companion enhanced their sense of safety during severe hypoglycemic episodes, as companions provided immediate assistance, including calling emergency services, during emergencies or incapacitation.

*If there’s no one else around, I definitely don’t feel secure. But if there is someone around, even if they aren’t a professional, they can call 120 for me.* (P11)

*I’ve heard that sugary drinks raise blood glucose faster. Conveniently, there’s a juice store right next door, so I can get one quickly if I need it.* (P15)

##### 3.2.3.3 Sub-theme 3: spiritual solace and psychological comfort

One participant reported that Buddhist prayer provided psychological comfort and enhanced her sense of confidence and safety.

*My mom took me to pray to the Buddha for my blessings, hoping that I would be safe and not have such severe hypoglycemia again. After praying to the Buddha for help, I feel a little bit calmer. Maybe it’s just to give myself some psychological comfort to feel supported.* (P15)

A sense of certainty about future outcomes may also affect the confidence of participants in managing hypoglycemic episodes and subsequent complications, potentially through positive psychological cues.

*My house is at the Cultural Park on Zhongshan Street, and there is a blind fortune-teller there. I asked him what would happen to me with diabetes, and he said I would live to 72 years old. Since then, I think I should have no problem dealing with hypoglycemia.* (P10)

#### 3.2.4 Theme 4: physiological and emotional arousal

Physiological and emotional states serve as key sources of self-efficacy information, determining how individuals interpret bodily signals as cues for competent action or as harbingers of threat. Participants who could recognize early warning signs, such as sweating or tremors, described a sense of security that was translated into confident management. Conversely, those who experienced severe hypoglycemic episodes or lived with fear of sudden onset hypoglycemia reported that these somatic and affective memories eroded their confidence, leaving them vigilant yet uncertain.

##### 3.2.4.1 Sub-theme 1: recognition and signs of hypoglycemia-related physical symptoms

Patients sensitive to hypoglycemic symptoms can adopt timely corrective measures, such as oral glucose supplementation and cessation of physical activity, to reverse hypoglycemia before experiencing adverse outcomes. The ability to detect such symptoms provides a sense of safety and enhances confidence in the prevention of severe hypoglycemic episodes.

*While I was working on the mountain, I felt a mild panic attack and I stopped working immediately. The feeling was fine, I knew it wasn’t serious and I just ate some food.* (P2)

*I’m very sensitive and when hypoglycemia happens, I can feel it right away.* (P5)

Some participants experienced prodromal sensations before reaching hypoglycemic thresholds. One participant reported that she perceived impending hypoglycemia before her blood glucose levels reached the hypoglycemic threshold, even in the absence of overt symptoms. Another participant reported hypoglycemia-related discomfort during sleep that awakened him. These prodromal warning signs can alert patients to implement timely interventions and prevent further decline in blood glucose levels.

*Now I’m so used to it that I can tell hypoglycemia is approaching if I feel like I’m about to go hypoglycemic, even though my blood sugar isn’t really that low at that point.* (P5)

Feeling panicky, when I sleep, I feel a bit uncomfortable. I wake up immediately, even if I am asleep at night in a dream. (P10)

Participants reported a spectrum of hypoglycemic symptoms, with the most prevalent symptoms being hunger, diaphoresis, fatigue, anxiety, dizziness, and somnolence. Less frequent manifestations included lingual paresis, irritability, and mood change.

*I was very emotional after one of my hypoglycemic episodes, and I easily got angry and became irritable.* (P2)

Asymptomatic hypoglycemia, insufficient knowledge, and negative experiences eroded the confidence of participants in taking timely corrective measures.

*In the morning, the nurse tested my blood sugar, which was slightly above 3 mmol/L. I realized I was hypoglycemic, but I didn’t feel anything.* (P3)

*I had a hypoglycemic episode one night, but I didn’t feel sick. About 12 pm, the nurse came to measure my blood glucose level and found that I was hypoglycemic*. (P8)

##### 3.2.4.2 Sub-theme 2: negative emotional arousal and lack of confidence

Most participants described sudden-onset hypoglycemic episodes in which blood glucose levels dropped rapidly, leaving insufficient time for timely responses. Thus, they were uncertain regarding the prevention of recurrent episodes and reported diminished confidence in the management of hypoglycemia.

*Hypoglycemic episodes are sometimes too sudden to manage. You never know when they will come, and you end up lacking confidence in handling them.* (P12)

As noted previously, severe hypoglycemic events exacerbated the anxiety and helplessness of participants, eroding their confidence in the management of hypoglycemia. For instance, one participant lost consciousness after experiencing two severe episodes of hypoglycemia within a single month and was transported to a hospital by an ambulance summoned by a family member.

*Just like the last two times, I was taken by ambulance. I had two hypoglycemic episodes in 1 month, during which I suddenly lost all my strength. Then, I collapsed, and I could have died without even realizing it.* (P4)

*The worst episode was the one over 10 years ago. I was building a house and it was very high. I felt hungry, and suddenly I couldn’t see. Then, I fell to the ground. Luckily, my husband was downstairs and he came up rapidly. He immediately brought me a bottle of sugar water and poured it into my mouth. After drinking it and lying there for a while, I opened my eyes and slowly got better.* (P1)

Potential life-threatening crises associated with hypoglycemia, such as falls, coma, and death, induced fear in participants. They personally experienced some of these severe adverse events and witnessed others experiencing similar outcomes. Due to these severe hypoglycemia-related experiences, they felt vulnerable and powerless, disrupting their daily life and work routines and undermining their confidence in the management of hypoglycemia.

It was pretty scary, the doctor said that hypoglycemia can lead to coma and even (death). It sounds scary to me. I didn’t know hypoglycemia was so scary before. (P13)

One participant who was engaged in height-related work was particularly concerned about safety risks associated with hypoglycemia.

*My hypoglycemic symptoms are not uniform. When working at heights, I get scared of falling, and it’s dangerous, but if I’m hyperglycemic, I don’t have that problem.* (P6)

Participants frequently faced a dilemma between hyperglycemia control and hypoglycemia prevention. These challenges eroded their confidence in managing hypoglycemia. One participant described concerns about hypoglycemia while fasting before gastroenteroscopy.

*What if I need to empty my bowels for a colonoscopy and can’t eat for an extended period?* (P8)

Another participant was concerned that corrective glucose intake following hypoglycemia might compromise overall glycemic control.

*When I was experiencing hypoglycemia, my blood sugar would increase as soon as I ate something.* (P1)

## 4 Discussion

This study provided the first systematic qualitative investigation of HC among Chinese adults with diabetes. Our findings indicated that HC is not a fixed trait but a dynamic construct continuously shaped through the interplay of four mechanisms: mastery experiences, vicarious learning, social persuasion, and physiological-emotional feedback. In other words, HC is a complex, agentic experience that can be enhanced through the synergistic interplay between the subjective efforts of patients and social environmental support. This process can enhance the well-being of patients, gradually developing confidence and security in the management of hypoglycemia, which in turn strengthens their management capacity.

### 4.1 The dual nature of HC: a dynamic construct shaped by mastery and vulnerability

The proactive and avoidant behaviors observed in this study reflect a fundamental tension in the self-management of diabetes. Those adopting proactive approaches demonstrated what Bandura (1977) termed resilient self-efficacy, the capacity to recover from setbacks and refine strategies through experiential learning. These participants transformed hypoglycemic episodes into opportunities to refine self-management regimens, thereby reinforcing glycemic control. Participants with adequate self-management competencies, such as blood glucose monitoring, may more easily recognize and address hypoglycemic symptoms in a timely manner, reducing the risks of complications and improving overall well-being (Lan et al., 2025a). Proactive Coping Theory suggests that individuals anticipate stress and take precautions to prevent it (Aspinwall and Taylor, 1997). Participants concerned about their diabetes acted more proactively in terms of glycemic control. Conversely, in disease and recovery periods, individuals may adopt various psycho-behavioral responses, including confrontation, avoidance, and submission (Feifel et al., 1987). Some participants refused to seek help and even ignored uncomfortable symptoms during hypoglycemic episodes. They appraised these events as threats, which triggered emotion-focused coping and defensive behaviors, such as insulin dose reduction or excessive food intake. This pattern is particularly concerning since it interferes with mastery experiences, further eroding confidence and leading to greater avoidance (Polonsky et al., 2020). Our data indicated that this pattern may be especially prevalent among Chinese patients due to cultural emphasis on self-reliance and reluctance to burden others, a theme further discussed in subsequent sections.

### 4.2 Symptom perception as the physiological foundation of HC: beyond simple awareness

Our findings position symptom perception as the physiological cornerstone of HC. First, intact hypoglycemia awareness relies on the counter-regulatory response system, in which glucagon and epinephrine release generate autonomic warning symptoms (sweating, tremors, palpitations) before the incidence of neuroglycopenia (Seaquist et al., 2022). Patients retaining this physiological capacity can modify declining glucose levels before the development of cognitive impairment. This establishes a temporal window for self-correction that reinforces confidence through successful self-rescue. This is consistent with the results of previous studies identifying hypoglycemia awareness as an important prerequisite for self-care (Puhr et al., 2019). Second, symptom recognition activates the belief that one can act before crisis escalation. Participant P5 detected impending hypoglycemia before reaching clinical thresholds. This anticipatory capacity transforms reactive management into proactive control, fundamentally altering the patient-hypoglycemia relationship from passive victimhood to active agency.

However, asymptomatic hypoglycemia disrupts both pathways. Specifically, the unpredictability of hypoglycemia undermines the predictability required for self-efficacy beliefs (Bandura, 1977). Nocturnal asymptomatic hypoglycemia is particularly insidious because it occurs during sleep and may lead to serious complications, including coma and death (Edelman and Blose, 2014). This is consistent with the results of previous studies identifying impaired awareness of hypoglycemia (IAH) as a key challenge in the management of hypoglycemia (Edelman and Blose, 2014; Lin et al., 2025; Seaquist et al., 2022), suggesting that IAH poses a significant threat to the maintenance of HC.

### 4.3 The Chinese cultural context and social construction of HC

The most distinctive contribution of this study lies in illuminating how Chinese cultural values uniquely configure the four sources of self-efficacy. Although previous Western studies have identified similar sources of efficacy (Polonsky et al., 2017b; Sönmez Sari et al., 2024), our data revealed culturally specific mechanisms modifying their operation in the Chinese context.

The cultural imperative of maintaining face and avoiding burdening others emerged as a critical barrier to the development of HC. Participant P12’ s refusal of immediate help during hypoglycemia and choosing to wait for her son reflects the deep-rooted cultural value of self-sufficiency and reluctance to impose a burden on non-family members. This “face-protective avoidance” preserves social dignity in the short term but delays effective intervention and interferes with patients’ mastery experiences that can build confidence. This finding extends previous Western literature on hypoglycemia avoidance by identifying a culturally specific mechanism known as face concern. It transforms a potentially adaptive social behavior into a maladaptive health behavior.

Unlike Western studies emphasizing individual autonomy in the management of diabetes (Polonsky et al., 2020), our participants constructed HC primarily through the family unit. Spousal monitoring, family emergency preparedness, and intergenerational knowledge transmission served as critical sources of verbal persuasion and vicarious learning in this study. This reflects the Chinese cultural model of interdependence (Konakawa et al., 2026), in which self-efficacy is not merely an individual attribute but a distributed property of the family system. We found that companionship can enhance confidence even when companions lacked medical expertise (P11). This underscores that social connection itself, rather than technical competence, provides the efficacy-enhancing function, a nuance overlooked in individualistic frameworks.

The integration of TCM practices (acupuncture, cupping, and herbal medicine) into the management of hypoglycemia represents a distinctively Chinese pathway to HC. Unlike Western patients, who typically relied solely on biomedical interventions, our participants integrated biomedical and traditional knowledge systems, establishing personalized management frameworks. Notably, following recurrent hypoglycemic episodes, some participants turned to TCM techniques, seeking to condition their bodies through holistic regulation. Those who successfully incorporated TCM into their routines reported not only physiological benefits (e.g., reduced frequency of hypoglycemia) but also enhanced confidence. They attributed these advantages to perceived holistic bodily regulation. This dual benefit reflects alignment among the holistic view of TCM, evidence-based care concepts, and the needs of some patients for gentle glycemic control (Pinto et al., 2021), suggesting that culturally embedded medical resources can provide additional support for the management of hypoglycemia. Consequently, HC in Chinese patients may be strengthened when interventions align with culturally embedded body concepts rather than relying exclusively on biomedical explanations. For instance, spleen deficiency serves as an illustrative TCM framework for understanding metabolic instability (Li et al., 2026). This finding challenges the cultural universality of existing HC interventions and supports the development of culturally congruent nursing protocols that legitimize the traditional health practices of patients.

Interestingly, some patients adopted culturally specific psychological adjustment strategies, such as Buddhist prayer and fortune-telling. Although these practices may appear irrational from a biomedical perspective, our analysis supported their efficacy-enhancing mechanisms. Fortune-telling (P10) provided a concrete future narrative (living to 72 years) and transformed the vague threat of mortality due to hypoglycemia into a bounded, manageable risk. Buddhist prayer (P15) operated through entrusting protection to a higher power, thereby reducing the psychological burden of sole self-responsibility and establishing emotional space for the development of confidence. These findings suggest that such spiritual practices serve as important sources of psychological comfort and emotional resilience, highlighting the critical role of spiritual support in the maintenance of HC among Chinese patients (Liu et al., 2026). Therefore, they may be recognized as culturally embedded coping resources rather than regarded as superstition.

## 5 Limitations

This study provided preliminary insights into HC experiences among Chinese patients with diabetes and explored its facilitators and barriers. However, since this was a qualitative study, these findings should be validated using quantitative research designs and standardized and objective assessment tools. Another limitation stems from the multi-ethnic composition of China, which encompasses diverse cultures and beliefs. This single-center study included participants from a single cultural background, lacking multicultural and cross-regional perspectives. Thus, future studies should explore facilitators and barriers to HC among patients with diabetes from multicultural perspectives to develop multifaceted, culturally tailored nursing intervention programs. Finally, we included only one participant with T1DM, which limited our ability to compare HC experiences between patients with T1DM and T2DM or generate T1DM-specific insights. Although the fundamental mechanisms involved in hypoglycemia and its management are similar across different types of diabetes, patients with T1DM typically experience more frequent and severe hypoglycemic episodes due to absolute insulin deficiency and longer disease duration. Therefore, the generalizability of our findings to patients with T1DM may be limited. Future studies should specifically target patients with T1DM to explore whether the identified facilitators and barriers operate differently in this population. Particularly, such studies should investigate the impact of recurrent severe hypoglycemia on confidence development.

## 6 Conclusion

This study provides the first qualitative exploration of HC among Chinese adults with diabetes, revealing that HC is dynamically shaped by four interrelated sources of self-efficacy information. Our findings suggested several implications for clinical practice. First, healthcare professionals may benefit not only from assessing whether patients have experienced hypoglycemia but also from evaluating how they respond to these experiences. Second, the integration of traditional Chinese medicine and spiritual practices into patients’ coping repertoires underscores the importance of culturally congruent care respecting indigenous health beliefs. Furthermore, asymptomatic hypoglycemia emerged as a particularly insidious threat to HC. Meanwhile, deficient knowledge of patients about the triggers of hypoglycemia and its pharmacological management suggests that existing patient education programs may not be sufficiently tailored to self-efficacy building. Future research should validate these qualitative findings through quantitative designs, develop culturally adapted HC measurement instruments for Chinese populations, and investigate interventions targeting the four sources of efficacy identified in this study. Finally, by shifting the conceptual focus from hypoglycemia as a source of fear to the management of hypoglycemia as a domain of self-efficacy, this study sought to guide the development of patient-centered, culturally sensitive interventions that empower individuals with diabetes to achieve both glycemic safety and psychological well-being.

## Data Availability

The original contributions presented in this study are included in this article/supplementary material, further inquiries can be directed to the corresponding author.

## Appendix

**APPENDIX 1:** Consolidated Criteria for Reporting Qualitative studies (COREQ).

Paper section/topic	Item	Description	Reported?	
				Pg.#
Domain 1: research team and reflexivity				
Personal characteristics				
Interviewer/facilitator	1	Which author/s conducted the interview or focus group?	√	P3
Credentials	2	What were the researcher’s credentials? e.g., Ph.D., MD	√	P3
Occupation	3	What was their occupation at the time of the study?	√	P3
Gender	4	Was the researcher male or female?	NA	
Experience and training	5	What experience or training did the researcher have?	√	P3
Relationship with participants				
Relationship established	6	Was a relationship established prior to study commencement?	√	P3
Participant knowledge of the interviewer	7	What did the participants know about the researcher? e.g., personal goals, reasons for doing the research	√	P3
Interviewer characteristics	8	What characteristics were reported about the interviewer/facilitator? e.g., bias, assumptions, reasons and interests in the research topic	√	P3
Domain 2: study design				
Theoretical framework				
Methodological orientation and theory	9	What methodological orientation was stated to underpin the study? e.g., grounded theory, discourse analysis, ethnography, phenomenology, content analysis	√	P2/P3
Participant selection				
Sampling	10	How were participants selected? e.g., purposive, convenience, consecutive, snowball	√	P3
Method of approach	11	How were participants approached? e.g., face-to-face, telephone, mail, email	√	P3
Sample size	12	How many participants were included in the study?	√	P3
Non-participation	13	How many people refused to participate or dropped out? Reasons?	√	P3
Setting				
Setting of data collection	14	Where was the data collected? e.g., home, clinic, workplace	√	P3
Presence of non-participants	15	Was anyone else present besides the participants and researchers?	√	P3
Description of sample	con	What are the important characteristics of the sample? e.g., demographic data, date	√	P4-5
Data collection				
Interview guide	17	Were questions, prompts, and guides provided by the authors? Was it used as a pilot test?	√	P3
Repeat interviews	18	Were repeat interviews carried out? If yes, how many?	NA	
Audio/visual recording	19	Did the research use audio or visual recording to collect the data?	√	P3
Field notes	20	Were field notes made during and/or after the interview or focus group?	√	P4
Duration	21	What was the duration of the interviews or focus group?	√	P3
Data saturation	22	Was data saturation discussed?	√	P3
Transcripts returned	23	Were transcripts returned to participants for comment and/or correction?	√	P3
Domain 3: analysis and findings				
Data analysis				
Number of data coders	24	How many data coders coded the data?	NA	
Description of the coding tree	25	Did the authors provide a description of the coding tree?	√	P5
Derivation of themes	26	Were themes identified in advance or derived from the data?	√	P4–9
Software	27	What software, if applicable, was used to manage the data?	√	P3
Participant checking	28	Did participants provide feedback on the findings?	NA	
Reporting				
Quotations presented	29	Were participant quotations presented to illustrate the themes/findings? Was each quotation identified? e.g., participant number	√	P4–9
Data and findings consistent	30	Was there consistency between the data presented and the findings?	√	P4–10
Clarity of major themes	31	Were major themes clearly presented in the findings?	√	P4–9
Clarity of minor themes	32	Is there a description of diverse cases or a discussion of minor themes?	√	P9–11

## References

[B1] Alberti K. G. Zimmet P. Z. (1998). Definition, diagnosis and classification of diabetes mellitus and its complications. Part 1: diagnosis and classification of diabetes mellitus provisional report of a WHO consultation. *Diabet Med* 15 539–553. 9686693 10.1002/(SICI)1096-9136(199807)15:7<539::AID-DIA668>3.0.CO;2-S

[B2] Alosaimi A. M. Alsulaimani N. H. Alotaibi W. A. (2022). Potential mechanisms for poor glycaemic control in patients with type two diabetes and fear of hypoglycaemia. *J. Diabetes Metab. Disord*. 21 1689–1697. 10.1007/s40200-022-01123-y 36404853 PMC9672273

[B3] American Diabetes Association Professional Practice Committee (2024a). 5. Facilitating positive health behaviors and well-being to improve health outcomes: standards of care in diabetes—2025. *Diabetes Care* S86–S127. 10.2337/dc25-S005 39651983 PMC11635047

[B4] American Diabetes Association Professional Practice Committee (2024b). 6. Glycemic goals and hypoglycemia: standards of care in diabetes-2024. *Diabetes Care* 47 S111–S125. 10.2337/dc24-S006 38078586 PMC10725808

[B5] Aspinwall L. G. Taylor S. E. (1997). A stitch in time: self-regulation and proactive coping. *Psychol. Bull*. 121 417–436. 10.1037/0033-2909.121.3.417 9136643

[B6] Bandura A. (1977). Self-efficacy: toward a unifying theory of behavioral change. *Psychol. Rev.* 84 191–215. 10.1037/0033-295X.84.2.191 847061

[B7] Bandura A. (1986). *Social Foundations of Thought and Action: A Social Cognitive Theory.* Hoboken, NJ: Prentice-Hall.

[B8] Demir I. Şahin G. Pamuk B. (2023). Validity and reliability of the hypoglycemia confidence scale for patients with type 1 diabetes. *Dahuder Med. J.* 3 76–84. 10.56016/dahudermj.1253273

[B9] Edelman S. V. Blose J. S. (2014). The impact of nocturnal hypoglycemia on clinical and cost-related issues in patients with type 1 and type 2 diabetes. *Diabetes Educ.* 40 269–279. 10.1177/0145721714529608 24695260

[B10] Feifel H. Strack S. Nagy V. T. (1987). Coping strategies and associated features of medically ill patients. *Psychosom. Med.* 49 616–625. 10.1097/00006842-198711000-00007 3423168

[B11] International Diabetes Federation [IDF] (2025). *Help Spread the Word about Diabetes and Well-Being. [Online].* Available online at: https://worlddiabetesday.org/get-involved/ (accessed November 14, 2026).

[B12] Khunti K. Alsifri S. Aronson R. Cigrovski B. M. Enters C. Forsén T. et al. (2016). Rates and predictors of hypoglycaemia in 27 585 people from 24 countries with insulin-treated type 1 and type 2 diabetes: the Global HAT Study. *Diabetes Obes. Metab.* 18 907–915. 10.1111/dom.12689 27161418 PMC5031206

[B13] Konakawa H. Kawai T. Tanaka Y. Hatanaka C. Li Q. Koh A. (2026). Examining the association between cultural self-construal and dream structures in China, Japan, and the United States. *Front. Psychol.* 16:1688407. 10.3389/fpsyg.2025.1688407 41602635 PMC12832506

[B14] Lan X. Zheng S. Ji X. Chen L. Zhou L. Ye B. et al. (2025a). Insulin injection knowledge, attitude, and practice of community nurses in a mountainous area of Southwest Zhejiang in China: a multicenter cross-sectional study. *Front. Endocrinol.* 16:1501992. 10.3389/fendo.2025.1501992 40900896 PMC12399374

[B15] Lan X. Zheng S. Li M. Lu S. Lou Y. Lian Z. et al. (2025b). Translation and validity of the Chinese version of the hypoglycemia confidence scale in adults with type 2 diabetes using both basal and prandial insulins: a psychometric analysis with cultural adaptation. *Diabetes Technol. Obes. Med*. 1 299–307. 10.1089/dtom.2025.0028

[B16] Li R. Dong P. T. Gao Y. B. Li A. Y. Wang Z. (2026). Zhongguo Zhong yao za zhi = Zhongguo zhongyao zazhi = China journal of Chinese materia medica. *Zhongguo Zhong Yao Za Zhi* 51 1172–1183. 41814838 10.19540/j.cnki.cjcmm.20251110.501

[B17] Lin Y. K. Ye W. Hepworth E. Rickels M. R. Amiel S. A. Speight J. et al. (2025). Impaired awareness and symptomatology of hypoglycemia in adults with type 1 diabetes using continuous glucose monitoring. *J. Clin. Endocrinol. Metab.* 110 e2553–e2561. 10.1210/clinem/dgae846 39657003 PMC12261081

[B18] Lincoln Y. S. Guba E. G. (2016). *The Constructivist Credo.* Abingdon: Routledge.

[B19] Lind M. Ólafsdóttir A. F. Hirsch I. B. Bolinder J. Dahlqvist S. Pivodic A. et al. (2021). Sustained intensive treatment and long-term effects on HbA1c reduction (SILVER Study) by CGM in people with type 1 diabetes treated with MDI. *Diabetes Care* 44 141–149. 10.2337/dc20-1468 33199470

[B20] Liu Z. Song D. Song Y. Liu Z. (2026). Religious exposure and concern for others’ health: religious priming promotes health-oriented altruism in nonreligious individuals through awe in China. *J. Relig. Health* 63. 10.1007/s10943-026-02642-1 41912965

[B21] Mandrik O. Severens J. L. Doroshenko O. Pan’kiv V. Kravchun N. Vlasenko M. et al. (2013). Impact of hypoglycemia on daily life of type 2 diabetes patients in Ukraine. *J. Multidiscip. Healthc.* 6 249–257. 10.2147/JMDH.S39133 23874102 PMC3712743

[B22] Menekli T. Şentürk S. (2022). The relationship between Hypoglycemic Confidence and hypoglycemia fear in type 2 diabetes mellitus patients. *Karya J. Health Sci.* 3 85–92. 10.52831/kjhs.1097901

[B23] Mesa ÁM. Belmonte S. Rodríguez P. Llanes M. V. Mateo C. Hidalgo L. et al. (2025). Fear of hypoglycemia is linked to poorer glycemic control and reduced quality of life in adults with type 1 diabetes. *Front. Endocrinol.* 16:1563410. 10.3389/fendo.2025.1563410 40453585 PMC12122291

[B24] Ogle G. D. Wang F. Haynes A. Gregory G. A. King T. W. Deng K. et al. (2025). Global type 1 diabetes prevalence, incidence, and mortality estimates 2025: results from the International diabetes Federation Atlas, 11th Edition, and the T1D Index Version 3.0. *Diabetes Res. Clin. Pract.* 225:112277. 10.1016/j.diabres.2025.112277 40412624

[B25] Pinto J. W. Bradbury K. Newell D. Bishop F. L. (2021). Lifestyle and health behavior change in traditional acupuncture practice: a systematic critical interpretive synthesis. *J. Altern. Complement. Med*. 27 238–254. 10.1089/acm.2020.0365 33332183

[B26] Polonsky W. (2020). Worries and concerns about hypoglycemia in adults with type 1 diabetes: an examination of the reliability and validity of the hypoglycemic attitudes and behavior scale (HABS). *J. Diabetes Complicat.* 34:107606. 10.1016/j.jdiacomp.2020.107606 32354623

[B27] Polonsky W. H. Fisher L. Hessler D. Edelman S. V. (2015). Identifying the worries and concerns about hypoglycemia in adults with type 2 diabetes. *J. Diabetes Complicat.* 29 1171–1176. 10.1016/j.jdiacomp.2015.08.002 26338296

[B28] Polonsky W. H. Fisher L. Hessler D. Edelman S. V. (2017a). Investigating hypoglycemic confidence in type 1 and type 2 diabetes. *Diabetes Technol. Ther.* 19 131–136. 10.1089/dia.2016.0366 27997217

[B29] Polonsky W. H. Fortmann A. L. Johnson K. E. Nguyen A. Beebe C. (2020). Hypoglycemic Confidence in the partners of adults with type 1 diabetes. *Diabetes Technol. Ther.* 22 249–255. 10.1089/dia.2019.0313 31638424

[B30] Polonsky W. H. Hessler D. Ruedy K. J. Beck R. W. Diamond Study Group. (2017b). The impact of continuous glucose monitoring on markers of quality of life in adults with type 1 diabetes: further findings from the DIAMOND randomized clinical trial. *Diabetes Care* 40 736–741. 10.2337/dc17-0133 28389582

[B31] Puhr S. Derdzinski M. Welsh J. B. Parker A. S. Walker T. Price D. A. (2019). Real-world hypoglycemia avoidance with a continuous glucose monitoring system’s predictive low glucose alert. *Diabetes Technol. Ther*. 21 155–158. 10.1089/dia.2018.0359 30896290 PMC6477579

[B32] Sandelowski M. (2010). What’s in a name? Qualitative description revisited. *Res. Nurs. Health* 33 77–84. 10.1002/nur.20362 20014004

[B33] Saunders A. L. Bodine C. Snell-Bergeon J. Forlenza G. P. Shah V. N. (2023). Higher prevalence of hypoglycemia and unsafe driving practices in adults with type 1 diabetes. *Diabetes Care* 46 e92–e93. 10.2337/dc22-2035 36706058

[B34] Saunders B. Sim J. Kingstone T. Baker S. Waterfield J. Bartlam B. et al. (2018). Saturation in qualitative research: exploring its conceptualization and operationalization. *Qual. Quant.* 52 1893–1907. 10.1007/s11135-017-0574-8 29937585 PMC5993836

[B35] Seaquist E. R. Teff K. Heller S. R. (2022). Impaired awareness of hypoglycemia in type 1 diabetes: a report of an NIDDK workshop in october 2021. *Diabetes Care* 45 2799–2805. 10.2337/dc22-1242 36455118 PMC9763030

[B36] Shi L. Fonseca V. Childs B. (2021). Economic burden of diabetes-related hypoglycemia on patients, payors, and employers. *J. Diabetes Complicat.* 35:107916. 10.1016/j.jdiacomp.2021.107916 33836965

[B37] Sönmez Sari E. Semerci Çakmak V. Çetinkaya, Özdemir S. Eşsiz Sefer B. (2024). Hypoglycaemic confidence levels and experiences related to the hypoglycaemia of patients with diabetes: a mixed methods study. *J. Clin. Nurs.* 33 1022–1035. 10.1111/jocn.17038 38284517

[B38] Strecher V. J. DeVellis B. M. Becker M. H. Rosenstock I. M. (1986). The role of self-efficacy in achieving health behavior change. *Health Educ. Q.* 13 73–92. 10.1177/109019818601300108 3957687

[B39] Tong A. Sainsbury P. Craig J. (2007). Consolidated criteria for reporting qualitative research (COREQ): a 32-item checklist for interviews and focus groups. *Int. J. Qual. Health Care* 19 349–357. 10.1093/intqhc/mzm042 17872937

[B40] Vears D. F. Gillam L. (2022). Inductive content analysis: a guide for beginning qualitative researchers. *Focus Health Prof. Educ* 23:1. 10.11157/FoHpe.v23i1.544

[B41] Ward A. Alvarez P. Vo L. Martin S. (2014). Direct medical costs of complications of diabetes in the United States: estimates for event-year and annual state costs (USD 2012). *J. Med. Econ.* 17 176–183. 10.3111/13696998.2014.882843 24410011

[B42] Zabin L. M. Qaddumi J. Ghawadra S. F. Battat M. M. (2025). Job stress and patient safety culture: a qualitative study among hospital nurses in Palestine. *BMC Nurs.* 24:308. 10.1186/s12912-025-02993-2 40128807 PMC11934625

